# Learning engagement and academic performance in Chinese higher education: serial mediation of grit and happiness and the conditional indirect effect of academic burnout

**DOI:** 10.3389/fpsyg.2026.1790839

**Published:** 2026-04-10

**Authors:** Zongzhe Wang, Zonghe Wang

**Affiliations:** 1 Guangdong Polytechnic of Industry and Commerce, Guangzhou, China; 2 Guangdong University of Finance and Economics, Guangzhou, China

**Keywords:** academic burnout, academic performance, Chinese university student, grit, happiness, learning engagement

## Abstract

This study aimed to examine whether grit and happiness mediate the relationship between learning engagement and academic performance, and whether this indirect effect is moderated by academic burnout among Chinese university students. This study employed a cross-sectional correlational survey design to examine the relationships among learning engagement, grit, happiness, academic burnout, and academic performance. A total of 656 undergraduate students were recruited using a multistage stratified sampling design across multiple regions in China. Data were collected using self-reported questionnaires and analyzed using SPSS 25.0, AMOS 23.0, and PROCESS Macro 4.2. Statistical techniques included descriptive statistics, reliability analysis, exploratory and confirmatory factor analyses, correlation analysis, measurement model testing, and moderated mediation analysis. The findings revealed that learning engagement, grit, happiness, and academic performance were positively and significantly correlated, whereas academic burnout was negatively correlated with each of these variables. Second, a moderated serial mediation effect was observed. Academic burnout moderated the indirect pathway from learning engagement to academic performance via grit and happiness. The conditional indirect effects were significant across low, mean, and high levels of academic burnout (effects ranging from.0110 to.0137; 95% bootstrap CIs did not include zero). These results highlight the mechanism of academic burnout as a psychological risk factor that can weaken the positive pathway from learning engagement to academic performance. The findings provide practical implications for educational practice, suggesting that interventions aimed at enhancing learning engagement, strengthening students' psychological resources (e.g., grit and happiness), and reducing academic burnout may help support students' academic success.

## 1 Introduction

In contemporary higher education, learning engagement has emerged as a crucial determinant of student success and academic performance. It encapsulates students' behavioral, emotional, and cognitive investment in learning activities (Fredricks et al., 2004). A growing body of research has demonstrated that engaged students are more likely to persist in challenging tasks, apply deeper learning strategies, and perform better academically (Ketonen et al., 2026; Song and Wang, 2026).

However, while the direct relationship between learning engagement and academic performance has been relatively well established, the psychological mechanisms that underlie this relationship remain underexplored. Specifically, recent advances in educational psychology suggest that non-cognitive factors such as grit and happiness may serve as critical mediators in translating engagement into measurable achievement outcomes (Li J. et al., 2025; Cheng and Zhang, 2023). Grit, defined as the perseverance and passion for long-term goals, has been linked to enhanced wellbeing and purpose in academic pursuits (Park et al., 2024a).

Happiness is often discussed in relation to subjective wellbeing (SWB), a broader construct that encompasses individuals' evaluations of their lives. Within well-established wellbeing frameworks, such as SWB and PERMA, happiness is generally regarded as a component of wellbeing rather than a direct synonym. In particular, subjective wellbeing is commonly understood as comprising both affective experiences (positive and negative emotions) and a cognitive evaluation of one's life, referred to as life satisfaction. Accordingly, in the present study, happiness is operationalized using the Satisfaction With Life Scale (SWLS), which primarily captures the cognitive dimension of subjective wellbeing—namely, individuals' overall judgments about their life satisfaction.

Likewise, students' happiness, reflecting their subjective evaluation of life satisfaction and wellbeing, has been shown to facilitate academic motivation and performance (Zapata and Céspedes-Carreño, 2025). From this perspective, students who experience higher levels of life satisfaction and positive psychological functioning may be more likely to sustain engagement in learning activities and achieve better academic outcomes. Thus, examining the role of happiness in relation to learning engagement, grit, academic burnout, and academic performance may provide a more comprehensive understanding of the psychological processes underlying students' academic success. Despite the increasing recognition of grit and happiness as mediators, few studies have investigated their combined (i.e., double mediation) roles in the relationship between learning engagement and academic performance, particularly in the context of Chinese university students. Moreover, the extent to which these mediational pathways are conditioned by contextual risk factors such as academic burnout remains largely unaddressed. Academic burnout—characterized by emotional exhaustion, cynicism, and reduced efficacy—has been widely recognized as a critical factor undermining student wellbeing and engagement (Ketonen et al., 2026). However, the moderating role of academic burnout in weakening the indirect pathway from learning engagement to academic performance through grit and happiness has received limited empirical attention.

Despite the growing body of research on student engagement and academic outcomes, existing studies have often examined psychological resources such as grit, happiness, and burnout in isolation rather than considering how these factors interact within an integrated framework. This gap is particularly salient in East Asian contexts, such as China, where academic pressures are intense and students frequently face challenges in maintaining psychological resilience. Addressing this limitation requires a more comprehensive approach that captures both the positive psychological resources that support learning and the vulnerabilities that may undermine academic functioning.

To address this gap, the present study proposes and tests a moderated serial mediation model in which grit and happiness jointly mediate the relationship between learning engagement and academic performance, while academic burnout moderates the indirect effects. By integrating engagement, psychological resources, and academic strain within a single analytical framework, this study provides a more nuanced understanding of how students' motivational and emotional processes interact under conditions of academic pressure. In doing so, the study contributes not only to the literature on student engagement and wellbeing but also to broader theoretical discussions on the interplay between psychological resilience and academic stress, while offering practical insights for supporting students' academic success in higher education contexts.

## 2 Theoretical background

### 2.1 Relationship between learning engagement and academic performance

Learning engagement refers to the extent to which students are actively involved in academic activities both behaviorally and emotionally. It encompasses participation, enthusiasm, cognitive investment, and persistence in learning tasks (Fredricks et al., 2004). Rather than reflecting mere compliance with academic requirements, engagement represents a deeper form of involvement in the learning process that facilitates self-regulated learning and sustained academic effort.

Academic performance, in contrast, represents the outcomes of learning processes and is commonly reflected in indicators such as grades, GPA, or test performance. Although achievement is often associated with cognitive ability, research has increasingly emphasized the role of motivational and behavioral factors—such as effort, persistence, and engagement—in shaping students' academic outcomes (Lu et al., 2026; Zhu and Liu, 2026).

A growing body of empirical research suggests that learning engagement plays a critical role in predicting academic success. Studies in higher education contexts indicate that students who display higher levels of emotional and cognitive engagement tend to utilize more effective learning strategies and demonstrate stronger academic performance (Ketonen et al., 2026). Similarly, research has shown that academic self-concept and positive academic emotions can enhance students' engagement in learning activities through mechanisms such as self-efficacy, ultimately contributing to improved academic outcomes (Song and Wang, 2026). Other studies focusing on tertiary education have also highlighted the role of motivational factors, including reading motivation and self-efficacy, in strengthening the link between engagement and academic success (Long, 2026).

Taken together, these findings indicate that learning engagement functions as a multidimensional construct that supports students' academic development. When students are emotionally, behaviorally, and cognitively engaged in their learning activities, they are more likely to persist in challenging academic tasks and achieve higher levels of academic performance.

### 2.2 Dual mediating effects of grit and happiness

#### 2.2.1 Mediating effects of grit

In contemporary educational psychology, grit—defined as sustained effort and enduring passion toward long-term goals (Duckworth et al., 2007)—has emerged as an important psychological construct in understanding how students translate learning engagement into academic performance. Unlike short-term perseverance, grit reflects an individual's capacity to maintain consistent interest and effort despite challenges, thereby contributing to long-term academic persistence and resilience (Datu et al., 2023; Karlen et al., 2022). Although grit has often been conceptualized as a relatively stable personality trait, recent research suggests that it can also be shaped and strengthened through educational experiences and supportive learning environments. From this perspective, grit can be understood as a relatively stable yet malleable motivational resource that develops over time through sustained engagement in learning activities.

A growing body of empirical research suggests that learning engagement can foster the development of grit by strengthening students' motivational and psychological resources. Studies in higher education contexts indicate that students who are more actively engaged in learning activities are more likely to develop sustained effort and goal commitment. For instance, engagement supported by positive instructional environments—such as perceived teacher rapport—has been shown to predict higher levels of grit in language learning contexts (He, 2025). Similarly, research has demonstrated that learning engagement can enhance students' self-efficacy, which in turn contributes to the development of the consistency dimension of grit (Chen et al., 2026).

At the same time, grit has been widely recognized as a significant predictor of academic performance. Students who demonstrate greater perseverance and long-term commitment to their goals tend to maintain sustained effort in challenging academic situations, which can lead to improved academic outcomes. Empirical evidence suggests that grit can enhance academic performance both directly and indirectly through psychological mechanisms such as self-efficacy and educational expectations (Wu et al., 2026). In addition, cross-cultural research has shown that grit, together with emotional creativity, contributes to students' academic engagement and success by supporting adaptive emotional regulation under academic pressure (Chen et al., 2025).

Taken together, these findings indicate that grit functions as a key motivational resource that helps translate students' learning engagement into meaningful academic outcomes. In this study, grit is conceptualized as a relatively stable yet developmentally responsive motivational resource that may mediate the relationship between learning engagement and academic performance.

#### 2.2.2 Mediating effects of happiness

Happiness is commonly discussed within the broader framework of subjective wellbeing and generally refers to individuals' enduring sense of positive emotional functioning and life satisfaction (Diener et al., 1985). In educational contexts, happiness extends beyond momentary pleasure and reflects positive emotional states that support students' resilience, motivation, and cognitive functioning in academic settings (Ou et al., 2025; Predoiu et al., 2025).

A growing body of research suggests that learning engagement is closely associated with students' emotional wellbeing, including their experience of happiness. Studies conducted in higher education environments indicate that supportive academic contexts can enhance both engagement and students' positive emotional states. For example, research in post-pandemic university settings has shown that students who perceive stronger institutional and instructional support tend to report higher levels of both learning engagement and happiness, highlighting the role of nurturing learning environments in promoting emotional wellbeing and academic persistence (Zapata and Céspedes-Carreño, 2025). Similarly, studies examining engagement in higher education contexts have demonstrated that higher levels of work or learning engagement are associated with greater psychological wellbeing among students and academic staff (Bandar et al., 2025).

Happiness has also been linked to academic performance through several cognitive and motivational mechanisms. Empirical studies suggest that positive emotional states can enhance students' concentration, adaptive coping strategies, and persistence in academic tasks. For instance, research has shown that happiness, together with health-related behaviors, can mediate the relationship between socioeconomic factors and educational success, illustrating how positive affect contributes to students' academic functioning (Li X. et al., 2025). In addition, evidence from adolescent populations indicates that psychological wellbeing—including happiness—supports emotional stability and academic engagement, which subsequently promotes improved learning outcomes (Park, 2025).

Taken together, these findings suggest that happiness functions as an important affective mechanism linking students' engagement in learning with their academic performance. Rather than merely representing an outcome of academic success, happiness may actively facilitate students' motivation, cognitive processing, and persistence, thereby serving as a mediating factor that helps translate learning engagement into sustained academic performance.

#### 2.2.3 Effect of grit on happiness

Grit is defined as perseverance and sustained passion for long-term goals (Duckworth et al., 2007). Individuals with higher levels of grit tend to maintain consistent effort and interest despite obstacles, setbacks, and prolonged challenges. Such persistence enables individuals to sustain engagement in goal-directed activities and increases the likelihood of achieving meaningful academic and personal outcomes.

From a theoretical perspective, grit may contribute to happiness through mechanisms related to goal pursuit and perceived progress toward valued goals. Goal progress theory suggests that perceiving meaningful progress toward personally significant goals promotes positive affect and enhances subjective wellbeing. Persistent effort toward long-term goals—a defining characteristic of grit—may therefore foster feelings of competence, mastery, and purpose, which in turn contribute to greater happiness and life satisfaction. In this sense, grit can be understood not only as a motivational trait that sustains effort but also as a psychological resource that facilitates positive emotional experiences.

Empirical research has increasingly supported the positive association between grit and wellbeing outcomes. Studies have consistently shown that individuals with higher levels of grit tend to report greater life satisfaction, stronger purpose in life, and higher levels of subjective wellbeing across different cultural contexts (Hill et al., 2016; Datu et al., 2018). Within educational settings, grit has also been linked to adaptive coping, resilience, and emotional stability, which are important psychological factors supporting students' wellbeing.

More recent studies focusing on university populations further highlight the role of grit in promoting students' happiness and emotional wellbeing. Research indicates that higher academic grit is associated with increased happiness and wellbeing, partly through cognitive and self-regulatory processes such as executive functioning and emotional regulation (Liao and Chen, 2022; Park et al., 2024b). In addition, evidence suggests that grit may reduce negative psychological outcomes, such as depression, while simultaneously enhancing life satisfaction and emotional stability among students (Liu Y. et al., 2022). Similarly, other studies have found that students with stronger perseverance toward meaningful academic goals tend to experience higher levels of happiness and psychological fulfillment (Cheng and Zhang, 2023).

Taken together, these theoretical perspectives and empirical findings suggest that grit can promote happiness by supporting sustained motivation, psychological resilience, and perceived progress toward meaningful goals. Through persistent goal pursuit, individuals may experience stronger feelings of competence, accomplishment, and purpose, which foster positive emotional states. Based on this reasoning, grit is expected to precede happiness in the proposed mediation framework. Accordingly, the present study examines whether grit and happiness sequentially mediate the relationship between learning engagement and academic performanceamong Chinese university students.

### 2.3 Moderating effect of academic burnout on the relationship between learning engagement and grit

Recent research suggests that learning engagement, grit, and academic burnout are closely interrelated constructs with important implications for students' motivation and academic functioning. Understanding how academic burnout moderates the relationship between learning engagement and grit requires examining the theoretical and empirical relationships among these variables.

A growing body of evidence indicates that learning engagement is negatively associated with academic burnout. Students who actively participate in learning activities and invest cognitive and emotional effort in their studies tend to experience lower levels of emotional exhaustion and academic cynicism. For example, research conducted among Chinese undergraduates in blended learning environments found that higher levels of learning engagement were associated with reduced academic burnout, particularly when institutional support was perceived to be strong (Fu and Qiu, 2024). Similar patterns have been observed in other educational contexts, where students demonstrating stronger academic engagement reported lower levels of emotional exhaustion and depersonalization, two core dimensions of academic burnout (Gichomo et al., 2024).

At the same time, academic burnout may undermine students' ability to sustain long-term effort and commitment, thereby weakening grit. Burnout is characterized by emotional exhaustion, cynicism toward academic tasks, and reduced academic efficacy, all of which may erode the motivational resources necessary for perseverance. Empirical studies have shown that burnout can diminish students' persistence and goal commitment, indirectly reducing levels of grit (Kim, 2020). Additional research has similarly demonstrated that higher levels of academic burnout are associated with lower levels of grit among university students, although social support may buffer this negative effect (Kim and Lee, 2022).

Taken together, these findings suggest that academic burnout may function as an important contextual factor shaping the relationship between learning engagement and grit. Although learning engagement typically promotes perseverance and sustained academic effort, students experiencing high levels of burnout may lack the emotional and cognitive resources required to translate engagement into long-term commitment. In contrast, in low-burnout contexts, the motivational benefits of engagement are more likely to strengthen students' perseverance and passion for academic goals.

Based on this reasoning, academic burnout is expected to negatively moderate the relationship between learning engagement and grit. Furthermore, considering the proposed mediation pathway linking learning engagement to academic performance through grit and happiness, the present study examines whether academic burnout conditions this indirect effect. Specifically, this study tests a moderated serial mediation model in which academic burnout attenuates the indirect influence of learning engagement on academic performance through grit and happiness among Chinese university students.

### 2.4 Hypotheses

Drawing on the theoretical framework and empirical literature discussed above, this study proposes a moderated mediation model linking learning engagement, grit, happiness, academic burnout, and academic performance among Chinese university students. Specifically, learning engagement is expected to influence academic performance through the sequential mediating roles of grit and happiness, while academic burnout is proposed to moderate this indirect pathway.

H1. Learning engagement is negatively associated with academic burnout and positively associated with grit, happiness, and academic performance.

H2. Academic burnout moderates the indirect effect of learning engagement on academic performance through grit and happiness.

## 3 Research methods

### 3.1 Research model

Based on prior empirical and theoretical research, this study proposes a moderated serial mediation model to examine whether academic burnout moderates the indirect effect of learning engagement on academic performance through grit and happiness.

**Figure 1:**
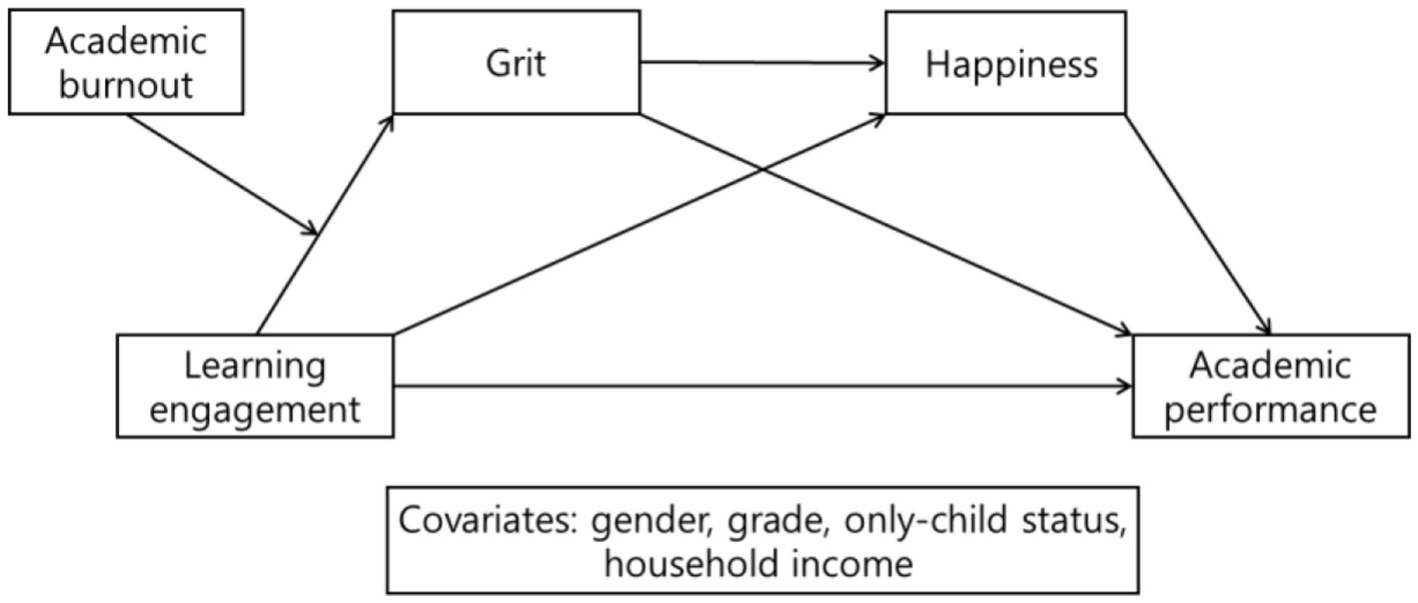
Conceptual research framework.

To test this moderated mediation framework, Model 83 of the SPSS PROCESS macro (Hayes, 2017) was employed. In the model, learning engagement was specified as the independent variable, academic performance as the dependent variable, and grit and happiness as sequential mediators. Academic burnout was included as a moderator of the path from learning engagement to grit, thereby testing whether the strength of this relationship varies depending on the level of burnout. Additionally, gender, grade, only-child status, and household income were statistically controlled to account for potential confounding effects. The conceptual framework is visually presented in Figure 1, illustrating the hypothesized relationships among the variables.

### 3.2 Participants and data collection method

This study employed a cross-sectional correlational survey design to investigate the relationships among learning engagement, grit, happiness, academic burnout, and academic performance among Chinese undergraduate students.

To recruit participants from diverse geographic regions, this study employed a multistage sampling procedure. First, five provinces were selected across China to represent the eastern, western, southern, northern, and central regions. From each selected province, one city was randomly chosen. In the next stage, one university was randomly selected within each city. Within each university, one department was selected based on accessibility and administrative feasibility for survey administration.

To implement this sampling framework, data collection was outsourced to a professional research agency, S Research Institute. The agency was commissioned to execute both sampling and data collection strictly in accordance with the predefined methodological criteria. The Institute verified that all procedures were conducted rigorously and in full compliance with the established guidelines.

Before data collection commenced, all prospective participants received detailed information regarding the study's objectives, procedures, confidentiality safeguards, and their rights, including the right to withdraw at any time without penalty. Only individuals who provided online informed consent were included in the study. The research protocol was reviewed and approved by the university's Institutional Review Board (IRB: HS25-12-03).

Based on an anticipated target of 100 participants per university, 150 questionnaires were distributed at each site, yielding a total of 656 valid responses for the final analysis. Among the respondents, 31.3% were male and 68.7% were female, indicating a higher proportion of female participants. Regarding grade distribution, 68.8% were first year, 9.9% second year, 15.7% third year, and 5.6% fourth year, with the first year comprising the largest group. Regarding major, humanities and social sciences were 69.8%, and science, Engineering, agriculture and medicine were 30.2%.

### 3.3 Research tools

#### 3.3.1 Academic engagement

Academic engagement was measured using the scale originally developed by Schaufeli et al. (2002) and later adapted for Chinese university students by Fang et al. (2008). The instrument assesses students' engagement in learning through statements such as “I think learning is a very meaningful activity” and “I believe that learning is the primary task in college.” The scale consists of 15 items, with higher scores indicating higher levels of academic engagement.

To examine the construct validity of the instrument, both exploratory factor analysis (EFA) and confirmatory factor analysis (CFA) were conducted. The EFA results suggested a two-factor structure, and all items demonstrated factor loadings above.50, meeting commonly accepted criteria for factor adequacy.

Because the structural model in the present study conceptualizes academic engagement as an overarching construct, an additional second-order confirmatory factor analysis was conducted to examine whether the two lower-order factors reflected a higher-order latent factor. In hierarchical measurement models, a second-order factor explains the correlations among lower-order factors and is commonly used to test the conceptual integration of multidimensional constructs. The results indicated that the higher-order factor loaded significantly on both lower-order factors, supporting the interpretation that the two dimensions can be represented by a common higher-order construct of academic engagement.

The initial measurement model showed acceptable but not optimal fit indices. Therefore, modification indices were examined. However, to avoid purely data-driven adjustments, only theoretically justifiable error covariances were allowed based on similarities in item wording and content. After these theoretically grounded modifications were introduced, the revised model demonstrated an acceptable fit to the data: χ^2^ = 402.531 (df = 82), χ^2^/df = 4.909, GFI = 0.923, CFI = 0.950, TLI = 0.937, and RMSEA = 0.077. Although the χ^2^/df value was slightly higher than conventional recommendations, this statistic is known to be sensitive to sample size. Considering the overall pattern of fit indices, the model fit was regarded as acceptable.

Convergent validity and construct reliability were evaluated at the first-order factor level because the observed items load directly onto the first-order factors in hierarchical models. The average variance extracted (AVE) values were 0.545 for Factor 1 and 0.535 for Factor 2, both exceeding the recommended threshold of 0.50. Composite reliability (CR) values were 0.914 and 0.873, respectively, indicating strong construct reliability. In addition, Cronbach's α values were 0.913 and 0.886, and McDonald's ω values were 0.914 and 0.873. These results indicate satisfactory internal consistency and convergent validity of the academic engagement scale.

#### 3.3.2 Academic burnout

Academic burnout was measured using the scale revised by Wu et al. (2010), which was adapted from earlier academic burnout measures for Chinese university students. The instrument assesses students' burnout in academic contexts through items such as “I can devote myself to study energetically” and “Recently, I feel an inner emptiness and do not know what to do.” The scale consists of 16 items rated on a five-point Likert scale, with higher scores indicating higher levels of academic burnout.

To examine the construct validity of the scale, both exploratory factor analysis (EFA) and confirmatory factor analysis (CFA) were conducted. The EFA results supported a four-factor structure, and all items showed factor loadings greater than 0.50, meeting commonly accepted psychometric criteria for factor adequacy.

Because the structural model in this study conceptualizes academic burnout as an overarching construct, a second-order confirmatory factor analysis was further conducted to examine whether the four lower-order factors reflected a hierarchical structure represented by a higher-order latent factor. In hierarchical measurement models, a second-order factor accounts for the correlations among lower-order factors and is widely used to examine the conceptual integration of multidimensional psychological constructs. The results indicated that the higher-order factor loaded significantly on all four lower-order factors, suggesting that the four dimensions can be explained by a common higher-order construct representing academic burnout.

Some fit indices of the initial measurement model were slightly below the most stringent recommended thresholds. Therefore, modification indices were examined. However, rather than making purely data-driven adjustments, only theoretically justifiable error covariances were allowed based on similarities in item wording and content. After introducing these theoretically grounded modifications, the revised model demonstrated an acceptable fit to the data: χ^2^ = 181.251 (df = 47), χ^2^/df = 3.856, GFI = 0.958, CFI = 0.955, TLI = 0.936, and RMSEA = 0.066. The CFI and TLI values exceeded the recommended cutoff of 0.90, and the RMSEA value was below 0.08, indicating acceptable model fit. Although the χ^2^/df value was somewhat higher than conventional recommendations, the chi-square statistic is known to be sensitive to sample size; therefore, the overall model fit was considered acceptable.

Convergent validity and construct reliability were evaluated using average variance extracted (AVE), composite reliability (CR), Cronbach's α, and McDonald's ω at the first-order factor level. The AVE values ranged from 0.440 to 0.721. Although the AVE value for Factor 2 was slightly below the recommended threshold of 0.50, the composite reliability values ranged from 0.674 to 0.838, exceeding the recommended criterion of 0.60. According to the criteria proposed by Fornell and Larcker (1981), convergent validity can still be considered acceptable when the AVE is below 0.50 but the composite reliability exceeds 0.60. McDonald's ω values ranged from 0.674 to 0.838, and Cronbach's α values ranged from 0.666 to 0.837, indicating acceptable levels of internal consistency. Lower reliability values for some factors may be attributable to the small number of indicators per factor, which can attenuate reliability estimates (Hair et al., 2019). Overall, these results indicate that the academic burnout scale demonstrates acceptable convergent validity and construct reliability.

#### 3.3.3 Grit

Grit was assessed using the Short Grit Scale (Grit-S) developed by Duckworth and Quinn (2009) and translated into Korean by Seek (2020). The scale consists of two theoretically derived subdimensions: Consistency of Interest and Perseverance of Effort. Each subdimension contains four items, resulting in a total of eight items. Participants responded using a five-point Likert scale ranging from 1 (strongly disagree) to 5 (strongly agree), with higher scores indicating higher levels of grit.

To examine construct validity, both exploratory factor analysis (EFA) and confirmatory factor analysis (CFA) were conducted. The EFA results supported a two-factor structure consistent with the theoretical dimensions of grit, and all items demonstrated factor loadings greater than.50, satisfying commonly accepted psychometric criteria.

Because the structural model in the present study conceptualizes grit as an overarching construct, a second-order confirmatory factor analysis was conducted to examine whether the two lower-order dimensions reflected a hierarchical structure represented by a higher-order latent factor. In hierarchical measurement models, a second-order factor accounts for the correlations among lower-order factors and is widely used to test the conceptual integration of multidimensional psychological constructs (Kline, 2016). The results indicated that the higher-order factor loaded significantly on both lower-order factors, suggesting that the two dimensions can be represented by a common higher-order construct of grit.

Some fit indices of the initial measurement model were slightly below the most stringent criteria; therefore, modification indices were examined. However, to avoid purely data-driven model adjustments, only theoretically justifiable error covariances were allowed based on similarities in item wording and content. After introducing these theoretically grounded modifications, the revised model demonstrated an acceptable fit to the data: χ^2^ = 46.879 (df = 12), χ^2^/df = 3.907, GFI = 0.980, CFI = 0.984, TLI = 0.971, and RMSEA = 0.067. The CFI and TLI values exceeded the recommended threshold of 0.95, and the RMSEA value was below 0.08, indicating an acceptable model fit (Hu and Bentler, 1999).

Convergent validity and construct reliability were evaluated at the first-order factor level because observed indicators load directly onto the first-order latent variables in hierarchical measurement models. The average variance extracted (AVE) values were 0.578 for Factor 1 and 0.452 for Factor 2. Although the AVE value for Factor 2 did not exceed the commonly recommended threshold of 0.50, the composite reliability values were 0.872 and 0.620, respectively. According to the criteria proposed by Fornell and Larcker (1981), convergent validity can still be considered acceptable when the AVE is below 0.50 but the composite reliability exceeds 0.60.

Cronbach's α values were 0.875 and 0.617, and McDonald's ω values were 0.872 and 0.620, showing consistent reliability estimates across different indices. The relatively lower reliability values for Factor 2 may be attributable to the small number of indicators, as reliability estimates are known to be sensitive to the number of items included in a scale (Hair et al., 2019). Overall, these findings suggest that the grit scale demonstrates acceptable levels of internal consistency and convergent validity in the present sample.

#### 3.3.4 Happiness

Happiness was measured using the Satisfaction with Life Scale (SWLS) originally developed by ed Diener et al. (1985) and later adapted for Chinese populations by Zhang et al. (2004). The scale evaluates individuals' global cognitive judgments of their life satisfaction and subjective wellbeing. It consists of eight items, including statements such as “Overall, I am satisfied with myself” and “I feel that I am a valuable person.” Responses were recorded on a five-point Likert scale ranging from 1 (strongly disagree) to 5 (strongly agree), with higher scores indicating higher levels of happiness.

To examine construct validity, both exploratory factor analysis (EFA) and confirmatory factor analysis (CFA) were conducted. The EFA results suggested a two-factor structure, with all items demonstrating factor loadings greater than.50, satisfying commonly accepted psychometric criteria. Because the structural model in the present study conceptualizes happiness as a single overarching construct, a second-order confirmatory factor analysis was conducted to examine whether the two lower-order factors reflected a hierarchical structure represented by a higher-order latent factor. In hierarchical measurement models, a second-order factor explains the correlations among lower-order factors and is commonly used to evaluate the conceptual integration of multidimensional psychological constructs (Kline, 2016). The results indicated that the higher-order factor loaded significantly on both lower-order factors, suggesting that the two dimensions can be represented by a common higher-order construct of happiness.

Some fit indices of the initial measurement model were slightly below the most stringent recommended criteria; therefore, modification indices were examined. However, rather than introducing purely data-driven changes, only theoretically justifiable error covariances were allowed based on similarities in item wording and content. After introducing these theoretically grounded modifications, the revised model demonstrated an acceptable fit to the data: χ^2^ = 56.51 (df = 15), χ^2^/df = 3.767, GFI = 0.978, CFI = 0.982, TLI = 0.966, and RMSEA = 0.065. The CFI and TLI values exceeded the recommended threshold of 0.95, and the RMSEA value was below 0.08, indicating an acceptable model fit (Hu and Bentler, 1999).

Convergent validity and construct reliability were evaluated at the first-order factor level because observed indicators load directly onto first-order factors in hierarchical models. The average variance extracted (AVE) values were 0.556 for Factor 1 and 0.444 for Factor 2. Although the AVE value for Factor 2 was slightly below the recommended threshold of 0.50, the composite reliability values were 0.858 and 0.702, respectively. According to the criteria proposed by Fornell and Larcker (1981), convergent validity may still be considered acceptable when the AVE is below 0.50 but the composite reliability exceeds 0.60.

Cronbach's α values were 0.851 and 0.707, and McDonald's ω values were 0.858 and 0.702, indicating consistent reliability estimates across different indices. Taken together, these findings suggest that the happiness scale demonstrates acceptable levels of internal consistency and convergent validity in the present study.

#### 3.3.5 Academic performance

Academic performance was measured using the Academic Achievement Scale developed by Wang (2011). The scale consists of nine items designed to assess students' persistence and effort in completing academic tasks, including statements such as “I persist in overcoming difficulties in order to complete learning tasks” and “In order to complete learning tasks on time, I have increased my study time.” Responses were recorded on a five-point Likert scale, with higher scores indicating higher levels of academic performance.

To examine the construct validity of the scale, both exploratory factor analysis (EFA) and confirmatory factor analysis (CFA) were conducted. The EFA results supported a single-factor structure consistent with the theoretical conceptualization of academic performance, with all factor loadings exceeding 0.50 and satisfying commonly accepted psychometric criteria.

Based on these findings, a CFA was subsequently performed to further test the measurement model. The CFA confirmed the one-factor structure, with all nine items demonstrating standardized factor loadings above 0.50. The model demonstrated acceptable fit indices: χ^2^(4) = 61.307, p < 0.001; χ^2^/df = 3.222; GFI = 0.980; TLI = 0.975; CFI = 0.987; and RMSEA = 0.058. These indices indicate a satisfactory level of model fit according to commonly recommended criteria (Hu and Bentler, 1999).

Convergent validity and construct reliability were also evaluated. The average variance extracted (AVE) was 0.522 and the composite reliability (CR) was 0.969, both exceeding recommended thresholds (Fornell and Larcker, 1981). Internal consistency reliability was further supported by Cronbach's α of 0.908 and McDonald's ω of 0.996, indicating excellent reliability of the academic performance scale.

### 3.4 Common method bias test

To mitigate potential common method bias (CMB), both procedural and statistical remedies were implemented in accordance with methodological recommendations (Podsakoff et al., 2003, 2012). Procedurally, respondent anonymity was assured and participation was voluntary in order to reduce evaluation apprehension. In addition, measurement items from different constructs were presented in a randomized order to minimize potential consistency motifs and response pattern biases.

As an initial diagnostic test, Harman's single-factor test was conducted by entering all measurement items into an unrotated exploratory factor analysis. The results indicated that the first factor accounted for 37.24% of the total variance, which is below the commonly suggested threshold of 50% (Podsakoff et al., 2003). Although Harman's test has been criticized for its limited sensitivity in detecting method bias (Podsakoff et al., 2012), this result provides preliminary evidence that a single latent factor does not dominate the covariance structure among the measurement items.

To further examine potential common method variance, a series of confirmatory factor analyses (CFA) was conducted. Specifically, the hypothesized measurement structure was compared with an alternative model incorporating a latent common method factor linked to all observed indicators (Williams et al., 1989). The baseline diagnostic CFA model showed a moderate level of fit (χ^2^/df = 5.143, CFI = 0.758, TLI = 0.746, RMSEA = 0.080, RMR = 0.048). When the latent method factor was included, model fit improved across all indices (χ^2^/df = 4.019, CFI = 0.831, TLI = 0.815, RMSEA = 0.068, RMR = 0.035), yielding ΔCFI = +0.073 and ΔRMSEA = – 0.012. This improvement indicates that some degree of common method variance may be present in the data.

Importantly, these CFA models were conducted as diagnostic analyses to assess potential method bias rather than as tests of the study's primary measurement model. Considering the combined evidence from the procedural remedies, the Harman's single-factor test results, and the diagnostic CFA comparison, the findings suggest that although some level of common method variance may exist, it is unlikely to substantially distort the substantive relationships examined in this study. Nevertheless, the results should be interpreted with appropriate caution regarding the potential influence of common method variance.

### 3.5 Data analysis

Data were analyzed using IBM SPSS Statistics (Version 25.0), AMOS (Version 23.0), and the SPSS PROCESS macro (Version 4.2; Hayes, 2017). Frequency analyses, reliability testing (McDonald's ω), exploratory factor analysis (EFA), confirmatory factor analysis (CFA), and measurement model evaluations were conducted to assess the psychometric properties of the instruments. To examine potential common method bias, a common method factor analysis was also performed.

To test the moderated mediation effects, PROCESS Model 83 was employed, enabling the estimation of conditional indirect effects in the presence of a moderator. Bootstrapping with 5,000 resamples and 95% bias-corrected confidence intervals was used to determine the statistical significance of the effects. All independent variables and the moderator were mean-centered to reduce potential multicollinearity in the moderation and moderated mediation analyses. Conditional effects and conditional indirect effects were examined at three levels of the moderator: one standard deviation below the mean (M – 1SD), the mean (M), and one standard deviation above the mean (M + 1SD).

## 4 Research results

### 4.1 Correlation between main variables

A series of bivariate correlation analyses were conducted to examine the associations among the key study variables in Table 1. All variables demonstrated statistically significant correlations in the expected directions, with the exception of academic burnout, which showed negative correlations with all other variables. Specifically, learning engagement was negatively correlated with academic burnout and positively correlated with grit, happiness, and academic performance, providing support for H1.

**Table 1:** Descriptive statistics and correlations among study variables (*N* = 656).

Variable	1	2	3	4	5
1. Learning engagement	1				
2. Academic burnout	−0.692^**^	1			
3. Grit	0.632^**^	−0.529^**^	1		
4. Happiness	0.789^**^	−0.608^**^	0.613^**^	1	
5. Academic performance	0.684^**^	−0.676^**^	0.552^**^	0.615^**^	1
M	3.616	2.8009	3.5207	3.6425	3.4629
SD	0.6208	0.48263	0.53686	0.64713	0.55334
Skewness	0.126	−0.034	0.351	−0.122	−0.363
Standard Error of Skewness	0.095	0.095	0.095	0.095	0.095
Kurtosis	0.109	1.742	−0.083	0.042	2.213
Standard Error of Kurtosis	0.191	0.191	0.191	0.191	0.191

Values below the diagonal are pearson correlation coefficients.

^**^p < 0.01.

The correlation between happiness and learning engagement was relatively high (*r* =0.789, *p* < 0.01), raising concerns regarding potential multicollinearity. To further assess this issue, a multiple regression analysis was conducted with academic performance as the dependent variable and learning engagement, academic burnout, grit, and happiness as predictors. The Variance Inflation Factor (VIF) values indicated no multicollinearity problems, with learning engagement showing a VIF of 3.449, which is well below the commonly accepted threshold of VIF < 5, a range generally considered acceptable in prior methodological literature (Loxia, 2023; Quantifying Health, 2023).

To evaluate the normality assumption, skewness and kurtosis values were examined. All variables exhibited skewness values below 1 and kurtosis values below 3, satisfying widely accepted criteria for univariate normality (Kim, 2013; Hair et al., 1998).

### 4.2 Moderated mediation effect of academic burnout

**Figure 2:**
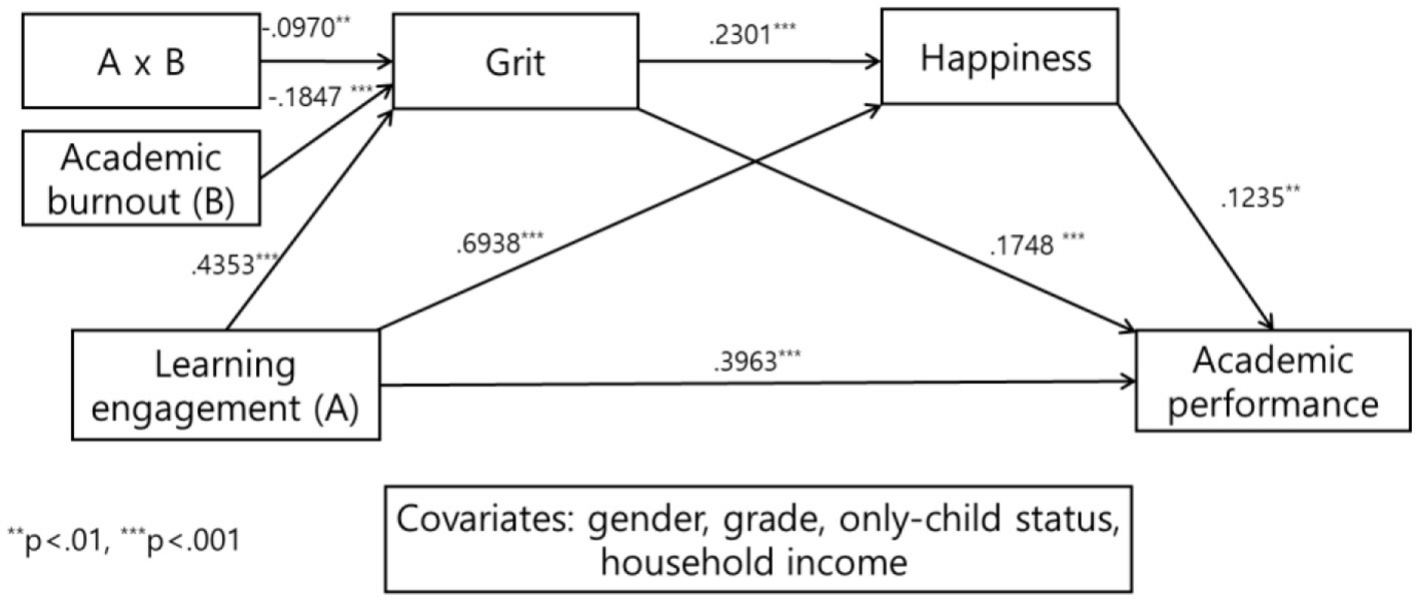
Statistical model of the moderated mediation effect of academic burnout.

**Figure 3:**
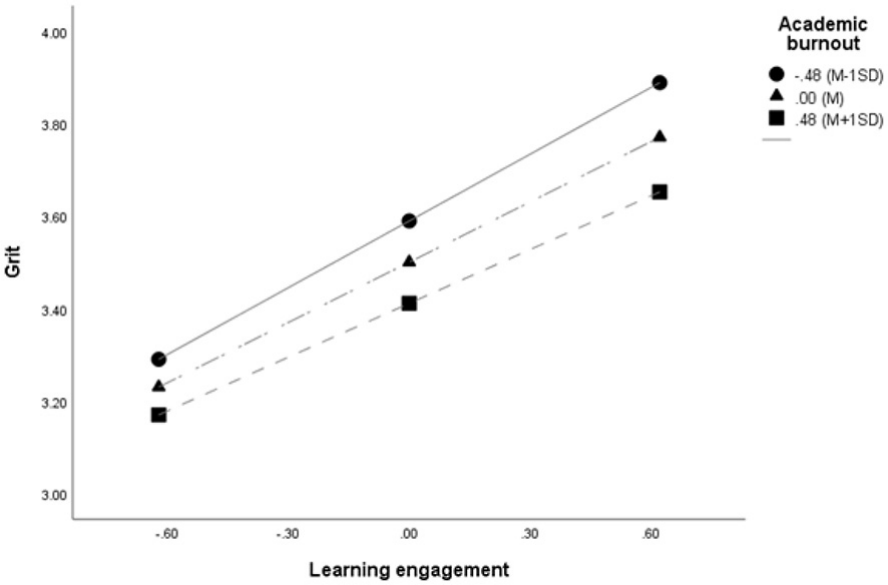
Moderating effect of academic burnout.

To examine whether academic burnout moderates the serial mediation effect of grit and happiness in the relationship between learning engagement and academic performance, PROCESS Macro Model 83 proposed by Hayes (2017) was employed. The results are presented in Figures 2, 3 and Tables 2, 3.

**Table 2:** Moderating effect of academic burnout in the moderated mediation model.

Predictor		DV: Grit			DV: Happiness			DV: Academic performance		
		B	SE	t	B	SE	t	B	SE	t
Constant		3.4982	0.0678	51.5783^***^	3.001	0.1412	21.2594^***^	2.3001	0.1872	12.2842^***^
IV	Learning engagement	0.4353	0.0359	12.1187^***^	0.6938	0.0315	21.9923^***^	0.3963	0.0424	9.3368^***^
M1	Grit				0.2301	0.0362	6.3561^***^	0.1748	0.038	4.6014^***^
M2	Happiness							0.1235	0.04	3.0882^**^
Moderator	Academic burnout	−0.1847	0.0463	−3.9865^***^						
Interaction	Learning engagement x Academic burnout	−0.097	0.0361	−2.6884^**^						
Higher order test	R^2^ change	0.0064								
	F	7.2274^**^								
Covariates	Gender	0.0049	0.0356	0.1368	−0.084	0.0334	−2.5191^*^	−0.0548	0.0341	−1.6065
	Grade	−0.0293	0.0171	−1.7132	−0.0332	0.0161	−2.067^*^	−0.0101	0.0164	−0.6182
	Only-child status	0.0409	0.0432	0.9447	−0.0394	0.0405	−0.9719	−0.024	0.0413	−0.5816
	Household income	0.0146	0.0209	0.6975	−0.0301	0.0195	−1.5499	0.0491	0.0198	2.4761^*^
**Model Summary**	R^2^	0.4261			0.6520			0.5071		
	F	68.7225^***^			202.6578^***^			95.2436^***^		
Mediating variable model (DV: Grit)										
Conditional effects of the learning engagement at values of the academic burnout										
Academic burnout		Effect	se		t		LLCI^*^		ULCI^**^	
−1 SD (Low)		0.4821	0.0394		12.2307^***^		0.4047		0.5595	
Mean		0.4353	0.0359		12.1187^***^		0.3647		0.5058	
+1 SD (High)		0.3885	0.0404		9.6137^***^		0.3091		0.4678	
Conditional effect of focal predictor at values of the moderator										
Academic burnout		Effect(B)	se		t		LLCI^*^		ULCI^**^	
−1.6343		0.5938	0.0681		8.7247^***^		0.4601		0.7274	
:										
2.0324		0.2382	0.0827		2.8814^**^		0.0759		0.4005	

B, unstandardized coefficient; SE, standard error; LLCI, lower limit of the 95% confidence interval; ULCI, upper limit of the 95% confidence interval.

^*^p < 0.05.

^**^p < 0.01.

^***^p < 0.001.

**Table 3:** Direct effect and conditional indirect effect.

Direct effect				
Learning engagement→Academic performance				
Effect	se	t	LLCI	ULCI
0.3963	0.0424	9.3368^***^	0.3129	0.4796
Conditional indirect effects				
Learning engagement→Grit→Happiness→Academic performance				
Academic burnout	Effect	BootSE	BootLLCI	BootULCI
−1 SD (Low)	0.0137	0.0069	0.0018	0.0291
Mean	0.0124	0.0063	0.0016	0.0267
+1 SD (High)	0.0110	0.0058	0.0014	0.0240

Boot SE, bootstrap standard error; BootLLCI, lower limit of the 95% bootstrap confidence interval; BootULCI, upper limit of the 95% bootstrap confidence interval.

^***^p < 0.001.

In the first mediation model, learning engagement had a significant positive effect on grit (M1) (B = 0.4353, *p* < 0.001). In contrast, the moderating variable, academic burnout, exerted a significant negative effect on grit (M1) (B = – 0.1847, *p* < 0.001). The interaction term also significantly and negatively predicted grit (M1) (B = – 0.0970, *p* < 0.01). Moreover, the change in R^2^ associated with the inclusion of the interaction term was significant (0.0064, *p* < 0.01). Therefore, academic burnout significantly moderated the relationship between learning engagement and grit (B = – 0.0970, *p* < 0.01), supporting H3.

Given the significance of the interaction term, conditional effects were examined. The conditional effects of learning engagement on grit at values of academic burnout (M – 1SD, M, and M + 1SD) were all significant. As academic burnout increased from M – 1SD to M + 1SD, the conditional effect decreased, indicating that academic burnout attenuated the positive influence of learning engagement on grit. Johnson–Neyman analysis further showed that the moderating effect was significant across the entire range of academic burnout, with the conditional effect of learning engagement decreasing as academic burnout increased.

Figure 3 illustrates this moderating pattern, showing a flatter slope for the high academic burnout group.

In the second mediation model, learning engagement had a significant positive effect on happiness (M2) (B = 0.6938, *p* < 0.001), and grit (M1) also significantly predicted happiness (M2) (B = 0.2301, *p* < 0.001).

In the dependent variable model, learning engagement had a significant positive effect on academic performance (B = 0.3963, *p* < 0.001), and grit (M1) also significantly predicted academic performance (B = 0.1748, *p* < 0.001). Additionally, happiness (M2) exerted a significant positive effect on academic performance (B = 0.1235, *p* < 0.01).

To examine the presence of a moderated mediation mechanism, both the direct effect and the conditional indirect effects were analyzed. The direct effect of learning engagement on academic performance was statistically significant and positive (B = 0.3963, SE = 0.0424, t = 9.3368, *p* < 0.001), indicating that higher levels of learning engagement are associated with improved academic performance.

Furthermore, the conditional indirect effects of learning engagement on academic performance through grit and happiness were estimated at three levels of academic burnout (M−1SD, M, M+1SD). Across all levels of the moderator, the bootstrapped 95% confidence intervals did not include zero. Notably, the magnitude of the conditional indirect effect decreased progressively as academic burnout increased from low (M−1SD) to high (M+1SD). The moderated mediation effect was significant, as the index of moderated mediation did not include zero (95% CI [LLCI, ULCI]). Therefore, H2 was supported.

## 5 Discussion and conclusion

The present study contributes to the growing body of literature on student engagement and academic outcomes by examining how psychological resources and vulnerabilities jointly shape academic functioning. Overall, the findings highlight the interconnected roles of learning engagement, grit, happiness, and academic burnout in predicting academic performance among university students.

Consistent with engagement theory and prior empirical research, learning engagement was positively associated with academic performance (Ketonen et al., 2026; Song and Wang, 2026). Engagement reflects students' active cognitive and emotional involvement in learning activities, which enhances persistence, attention, and the effective use of learning strategies. As previous studies have suggested, students who are more engaged in their academic work are more likely to invest sustained effort and derive meaning from their learning experiences, thereby achieving better academic outcomes.

The findings also demonstrated positive associations among grit, happiness, and academic performance. This pattern aligns with research emphasizing the role of non-cognitive psychological resources in supporting educational success (Liao and Chen, 2022; Liu H. et al., 2022). Grit, characterized by perseverance and sustained commitment toward long-term goals, has been linked to students' capacity to maintain effort despite academic challenges. At the same time, happiness—reflecting students' subjective evaluation of their wellbeing and life satisfaction—can foster positive emotions and adaptive coping strategies that support sustained motivation and performance. Together, these results suggest that motivational persistence and positive emotional functioning operate synergistically to facilitate academic performance.

In contrast, academic burnout was negatively associated with all other study variables, highlighting its detrimental influence on students' academic functioning. This finding is consistent with prior research showing that burnout undermines motivation, engagement, and psychological wellbeing in academic settings (Ou et al., 2025; Predoiu et al., 2025). When students experience emotional exhaustion, cynicism toward academic tasks, or feelings of inefficacy, their ability to remain cognitively involved in learning and to maintain positive emotional states may be substantially reduced. These patterns underscore the importance of considering both psychological strengths and stress-related vulnerabilities when examining students' academic outcomes.

Beyond these associations, the present study extends prior research by examining the conditional processes through which learning engagement contributes to academic performance. Specifically, the results revealed a significant moderated serial mediation model in which grit and happiness sequentially mediated the relationship between learning engagement and academic performance, while academic burnout weakened this indirect pathway. This finding provides empirical support for theoretical perspectives suggesting that burnout depletes students' motivational and emotional resources, thereby constraining the beneficial effects of engagement (Schaufeli and Taris, 2014).

More specifically, academic burnout moderated the relationship between learning engagement and grit, indicating that the positive influence of engagement on students' perseverance was attenuated when burnout levels were high. This pattern suggests that even when students are actively involved in learning activities, the presence of substantial academic stress and emotional exhaustion may hinder their ability to sustain long-term commitment and effort toward academic goals. In other words, burnout may function as a contextual constraint that disrupts the translation of engagement into psychological resources that ultimately support academic success.

Taken together, these findings provide a more nuanced understanding of how learning engagement translates into academic performance. While engagement is generally considered a protective factor in academic contexts, its effectiveness appears contingent upon students' psychological conditions, particularly the absence of severe burnout. By integrating engagement, psychological resources, and academic strain within a moderated serial mediation framework, this study contributes to a more comprehensive understanding of the dynamic processes underlying students' academic success.

These findings highlight important practical implications for higher education institutions seeking to support students' academic success and psychological wellbeing. Specifically, universities and educators should consider implementing multi-level intervention strategies that simultaneously strengthen students' psychological resources while reducing excessive academic pressure.

For example, instructors may incorporate learning strategies that actively promote student engagement and persistence, such as goal-setting activities, structured feedback, and opportunities for meaningful participation in learning tasks. At the institutional level, universities may develop programs aimed at fostering students' perseverance and long-term goal orientation in order to cultivate grit, while also creating supportive campus environments that enhance students' wellbeing and positive academic experiences.

In addition, student support services can play a critical role by providing academic counseling, stress management training, and psychological support programs designed to prevent or alleviate academic burnout. Such initiatives may help students maintain motivation and emotional balance even in academically demanding environments. Taken together, these findings suggest that effective educational interventions should not only promote engagement and psychological strengths but also address structural and psychological sources of academic stress in order to sustain students' academic performance and wellbeing.

In sum, the moderated mediation effect underscores a dual pathway: while learning engagement facilitates achievement via grit and happiness, this pathway is significantly weakened in the presence of high academic burnout. These insights contribute to a more nuanced model of student success that integrates both psychological assets and liabilities.

While this study provides important insights into the psychological mechanisms underlying the relationship between learning engagement and academic performance—particularly through the dual mediation of grit and happiness and the moderating effect of academic burnout—several limitations should be acknowledged.

First, the cross-sectional design of this study limits the ability to draw causal conclusions regarding the relationships among learning engagement, grit, happiness, academic burnout, and academic performance. Although the proposed moderated serial mediation model is theoretically grounded and statistically supported, the temporal ordering among these variables cannot be firmly established. Future research employing longitudinal or experimental designs would help clarify the causal direction and temporal dynamics underlying these relationships.

Second, the measurement of academic performance relied on self-reported behavioral indicators such as persistence and effort in academic tasks rather than objective performance indicators (e.g., GPA or examination scores). Although such indicators have been widely used in educational research to reflect students' academic functioning, the reliance on self-reported measures may introduce potential bias and inflate associations among related constructs. Future studies may benefit from incorporating objective academic records or multiple sources of assessment to provide a more comprehensive evaluation of academic performance.

Third, the conceptual proximity among some constructs examined in this study may also warrant caution in interpreting the strength of the observed relationships. For instance, behavioral indicators of academic performance share conceptual similarities with constructs such as learning engagement and grit. This overlap may partially contribute to the relatively strong associations observed among these variables. Future research could further disentangle these constructs by employing alternative measurement approaches or additional behavioral indicators.

Fourth, academic burnout was treated as a higher-order construct in the moderated mediation model to capture students' overall level of burnout. However, burnout is theoretically multidimensional, encompassing emotional exhaustion, cynicism, and reduced academic efficacy. Future research could investigate whether specific dimensions of academic burnout exert differential moderating effects within the mediation pathways, thereby offering a more nuanced understanding of its regulatory role.

Fifth, although participants were recruited using a multistage sampling procedure across several regions in China, the sample was restricted to Chinese university students. Consequently, the generalizability of the findings to other cultural or educational contexts may be limited. Cross-cultural studies could examine whether similar psychological mechanisms operate among students in different educational systems and sociocultural environments.

Sixth, participants were recruited from selected departments within each university, which may introduce potential cluster effects. Future research could adopt broader sampling strategies across multiple departments or institutions to reduce possible clustering bias.

Seventh, the gender distribution of the sample was unbalanced, with female students representing 68.7% of the participants. This imbalance may limit the generalizability of the findings across genders. Future studies should consider recruiting more gender-balanced samples to examine whether the observed relationships differ between male and female students.

Eighth, the cross-sectional nature of the data also leaves open the possibility of alternative or reciprocal relationships among the study variables. For example, students who achieve higher academic outcomes may subsequently experience greater happiness or become more engaged in learning activities. Future longitudinal research would be valuable in clarifying these potential reciprocal dynamics.

Finally, future studies could extend the present model by incorporating additional psychological mediators or moderators, such as academic resilience, growth mindset, or perceived social support. Examining how these factors interact with grit, happiness, and academic burnout may provide further insights into the complex psychological mechanisms underlying students' academic functioning.

## Data Availability

The original contributions presented in the study are included in the article/supplementary material, further inquiries can be directed to the corresponding author/s.
